# Diffuse large B cell lymphoma: using pathologic and molecular biomarkers to define subgroups for novel therapy

**DOI:** 10.1007/s00277-014-2116-y

**Published:** 2014-05-29

**Authors:** Antonino Carbone, Annunziata Gloghini, Yok-Lam Kwong, Anas Younes

**Affiliations:** 1 Department of Pathology, Centro di Riferimento Oncologico (CRO) Aviano, Istituto Nazionale Tumori, IRCCS, Via F. Gallini 2, 33081 Aviano, Italy; 2 Department of Diagnostic Pathology, Fondazione IRCCS Istituto Nazionale dei Tumori, Milano, Italy; 3 Department of Medicine, Queen Mary Hospital, Professorial Block, Hong Kong, China; 4 Lymphoma Service, Memorial Sloan Kettering Cancer Center, New York, NY 10065 USA

**Keywords:** DLBCL, Biomarkers, Prognosis, Diagnosis, Treatment

## Abstract

Diffuse large B cell lymphoma (DLBCL) comprises specific subtypes, disease entities, and other not otherwise specified (NOS) lymphomas. This review will focus on DLBCL NOS because of their prevalence and their heterogeneity with respect to morphology, clinical presentation, biology, and response to treatment. Gene expression profiling of DLBCL NOS has identified molecular subgroups that correlate with prognosis and may have relevance for treatment based on signaling pathways. New technologies have revealed that the “activated B cell” subgroup is linked to activation of the nuclear factor kB (NF-kB) pathway, with mutations found in *CD79A/B*, *CARD11*, and *MYD88*, and loss of function mutations in *TNFAIP3*. The “germinal center B cell-like” subgroup is linked to mutational changes in *EZH2* and *CREBBP*. Biomarkers that are related to pathways promoting tumor cell growth and survival in DLBCL have been recognized, although their predictive role requires clinical validation. Immunohistochemistry for detecting the expression of these biomarkers is a practical technique that could provide a rational for clinical trial design.

## Introduction

The application of advanced immunologic, biochemical, and genetic techniques in the classification of tumors of hematopoietic and lymphoid tissues has led to the identification of many distinct disease entities as proposed by the World Health Organization (WHO) working group [1]. Among tumors of lymphoid tissues, diffuse large B cell lymphoma (DLBCL) is the most common lymphoma, accounting for about 30 % of the cases, and comprises specific subtypes or disease entities. However, most cases are still classified as DLBCL, not otherwise specified (NOS) [2]. DLBCL NOS is a heterogeneous category with respect to morphology, clinical presentation, biology, and response to treatment. DLBCL can be subclassified based on cytologic appearance (e.g., centroblastic or immunoblastic morphology) and the site of primary involvement (nodal or extranodal) and according to the clinical background from which they arise (e.g., normal or compromised immunity) [3]. Importantly, these disparate features are reflected by the wide spectrum of clinical outcomes and treatment response, so that the development of new therapeutic strategies is urgently needed to address this heterogeneity in DLBCL [4, 5].

**Fig. 1:**
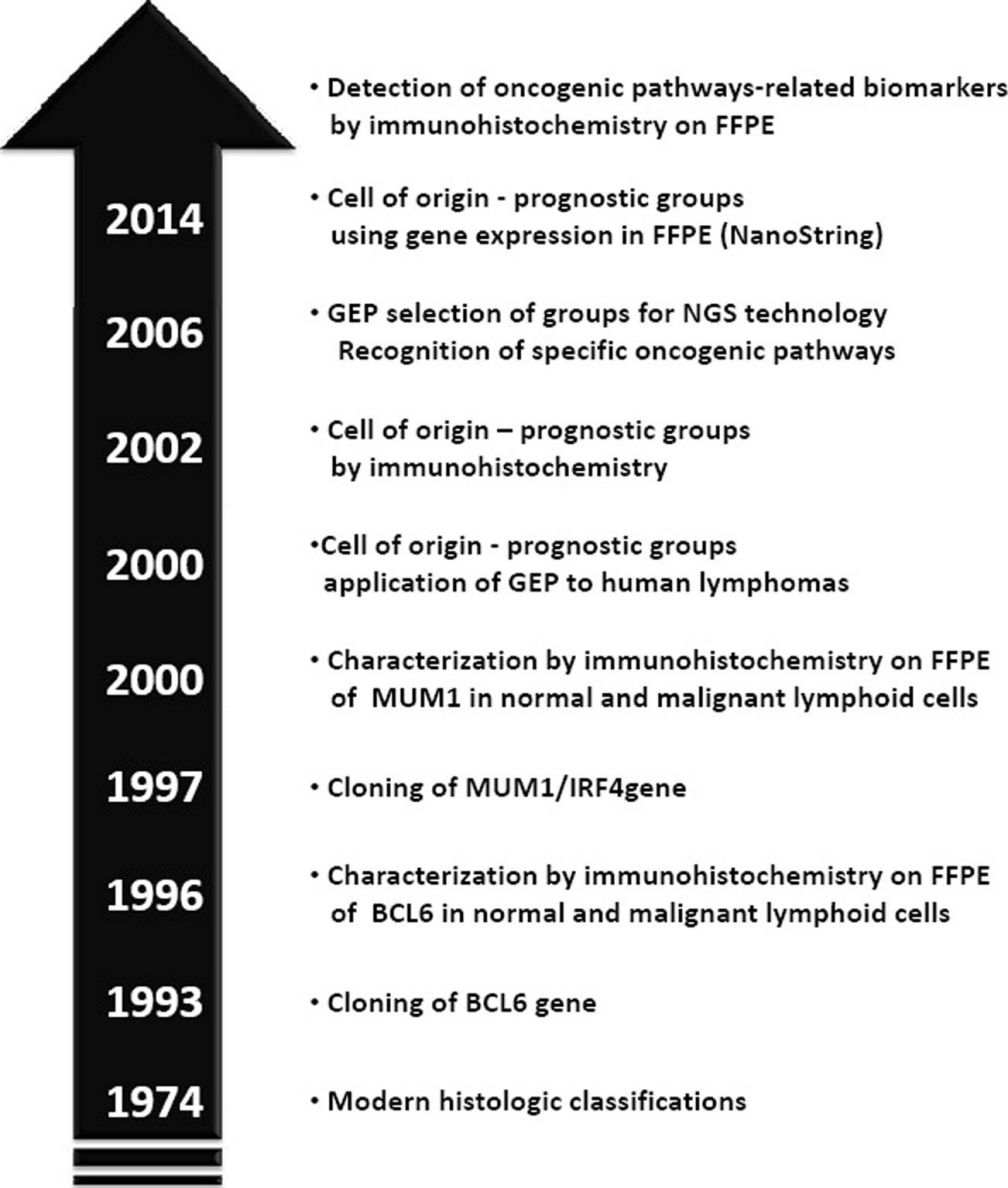
Evolution in the study of diffuse large B cell lymphoma focusing on prognosis and biomarkers detection

In recent years, gene expression profiling (GEP) studies identified gene expression signatures that can define the “cell of origin” of DLBCL cases. Subsequently, immunohistochemical signatures were developed to capture the distinction between the germinal center (GC) B-like DLBCL (GCB DLBCL) subgroup and the activated B-like DLBCL (ABC DLBCL) subgroup. Among the many advantages, GEP provides new information about the underlying molecular mechanisms of DLBCL and enables the grouping of more homogeneous subsets of patients with poorer prognosis who may benefit from improved treatments [6]. GEP also helps to select tumors for which next generation sequencing (NGS) technology may recognize specific oncogenic pathways, thus selecting patients who may benefit from identifiable therapeutic agents [6–9] (Fig. 1). However, an effective use of this information still requires reliable tests and treatment strategies that exploit these data.

This paper will focus on DLBCL, NOS, because of their prevalence and their heterogeneity. This is a synthetic review of the different biological abnormalities found in DLBCL, NOS, that reveal diagnostic or prognostic biomarkers. This review also describes discoveries that help to identify DLBCL subgroups and to recognize related oncogenic pathways, thereby providing a rationale for a more individualized approach in the treatment of this group of neoplasms.

## DLBCL classification

In 1994 the International Lymphoma study group [10] unified within a single “diffuse large B cell lymphoma” category, three high-grade lymphomas originally defined as “histiocytic” [11] and then, with updated terminology, named centroblastic lymphoma and its variants [12], immunoblastic B cell lymphoma [12, 13], and large B cell anaplastic Ki1+ lymphoma [14]. Notably, the DLBCL category included lymphoma subtypes that in a previous international classification (working formulation for clinical usage) [15], were split into two different prognostic groups (intermediate and high grade) [16]. Therefore, it was not surprising that important clinical studies showed that DLBCL NOS was heterogeneous with respect to clinical outcome [4]. Interestingly, the recent WHO classification proposal, in addition to specific DLBCL subtypes and disease entities, formally acknowledges the remaining DLBCL as DLBCL NOS. This category includes lymphomas with centroblastic, immunoblastic, and anaplastic morphology as common morphological variants [1]. The clinical relevance of these DLBCL variants remain debatable, although tumors composed predominantly of centroblasts have a better prognosis than those composed of immunoblasts [17]. Coherently, other studies have shown that immunoblastic morphology is highly significant in predicting an adverse outcome [18]. However, conclusions about the prognostic significance of these morphologic variants are hampered by poor reproducibility and a lack of consensus among pathologists [1–3].

## DLBCL subgrouping by cell of origin

**Fig. 2:**
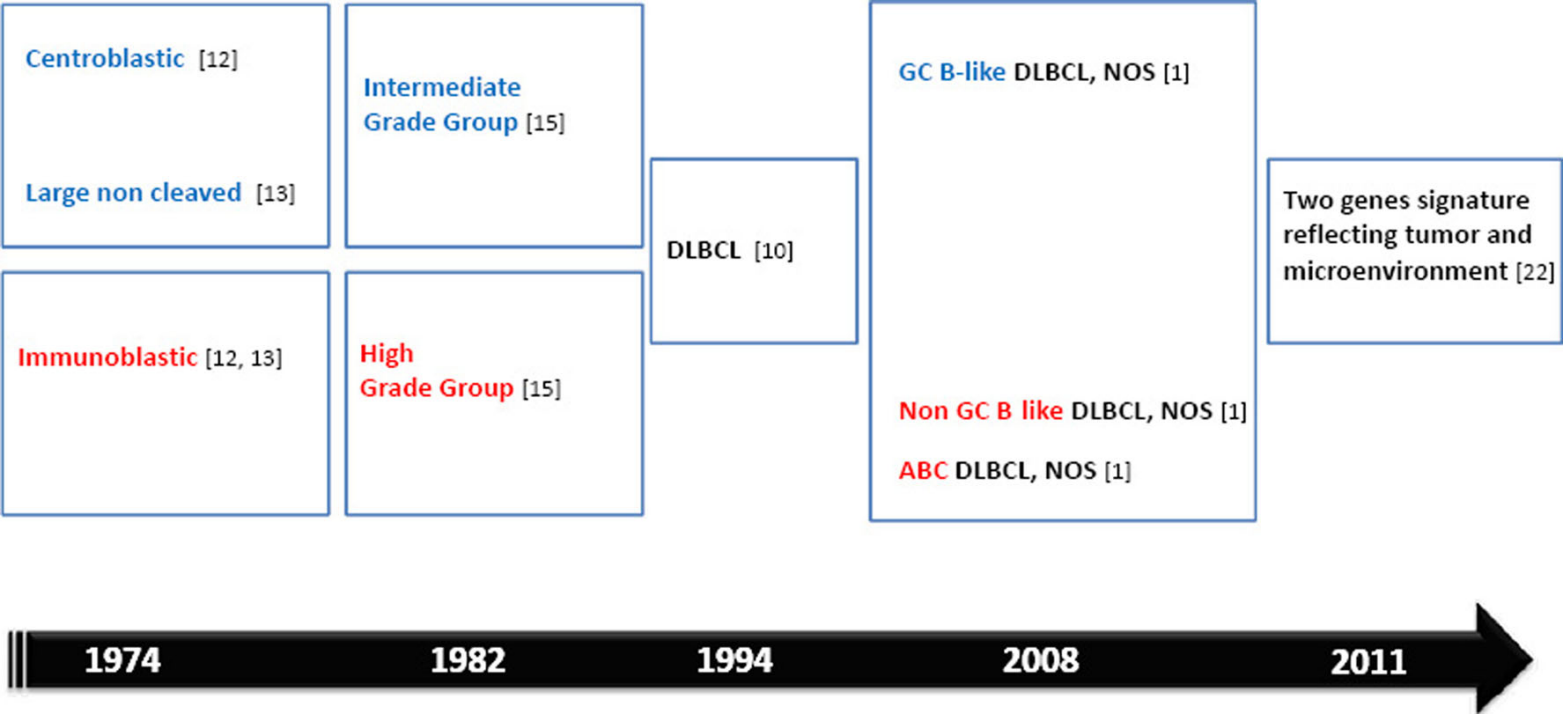
Diffuse large B cell lymphoma not otherwise specified. Subgroups with prognostic significance. *DLBCL* diffuse large B cell lymphoma, *NOS* not otherwise specified, *GC B-like* germinal center B cell like, *Non GCB-like* germinal center B cell like, *ABC* activated B cell

GEP of DLBCLs has identified molecular subgroups, which correlate with prognosis, and may have relevance for treatment based on signaling pathways (see below) [6–9, 19]. The original GEP studies have shown that at least two major subgroups of DLBCL could be identified. They resemble either germinal center B cells (GCBs) or activated B cells (ABCs), establishing a putative cell of origin [19]. GEP studies have demonstrated a significantly worse prognosis for the ABC subtype. The prognostic value of GEP has been independently validated by examining other selected gene sets [20, 21]. A six-gene model (favorable LMO2, BCL6, FN1; unfavorable CCND2, SCYA3, BCL2) was reported to identify approximately one third of DLBCL patients whose 5-year survivals were less than 27 % [21]. Furthermore, expressions of LMO2 and TNFRSF9 have been used to develop a two-gene signature based on tumor and microenvironment [22] (Fig. 2). In a large series of DLBCL patients, this simple model added prognostic value to the clinical International Prognostic Index (IPI) [22]. Very recently, a 20-gene gene expression assay in formalin fixed paraffin-embedded tissues has been proposed for the determination of cell of origin subgroups of DLBCL [23]. By this, assay identification of ABC versus GCB subgroups from paraffin-embedded tissue is now possible. In the validation cohort, these assays proved to be accurate and robust with a rapid turnaround time (Fig. 1).

Regarding the correlation between the morphologic variants of DLBCL NOS and GEP, a single study demonstrated that the immunoblastic subtype was enriched for cases with an ABC profile, whereas purely centroblastic neoplasms were more often GCB [24]. Consistently, an immunohistochemical study, performed in HIV-associated lymphomas, showed that the expression of BCL6 (a GC marker) and MUM1 (a post-GC marker) were mutually exclusive. BCL6 was generally restricted to the centroblastic and MUM1 to the immunoblastic variants of DLBCL [25]. However, cases co-expressing BCL6 and MUM1 were observed. They were considered as non-GC-related lymphomas (reviewed in [26]).

Because it is impractical to perform GEP involving mRNA expression in every case, various immunohistochemical profiles have been tested as surrogates [9, 27–31]. Although the correspondence was not exact, prognostic correlations were drawn with immunohistochemically defined groups. The first algorithm using CD10, MUM1, and BCL6 [27] did not appear to distinguish groups of significantly differing prognosis in almost all the series examined, when patients were treated with rituximab-containing regimens. Refinements of the approach have led to the incorporation of further markers including stromal response markers and microvessel density [32]. However, the concordance rate between the immunohistochemically defined and GEP-defined subgroups has been a variable [29, 30, 33, 34]. These observations raise the question whether the core of the original GEP and immunohistochemical algorithms still needs to be maintained. The question is still open, but it remains that MUM1 should be retained within the immunohistochemical profile together with BCL6, since it is a marker of transition from BCL6 positivity (GC B cells) to CD138 expression (immunoblasts and plasma cells) [35].

Conflicting results have been found in HIV-associated DLBCL [36, 37]. In particular, the role of molecular subgroups in predicting outcome in HIV-associated DLBCL is still unclear. A recent study aiming at understanding the role of oncogenic pathway-related biomarkers has shown that the molecular pathogenesis of immunodeficiency-associated lymphomas differs from that of lymphomas of the immunocompetent host with similar histology [38]. Immune deregulation, viral infections, and chronic antigenic stimulation may provide alternative survival signals that render neoplastic B cells less dependent on genetic lesions otherwise important for lymphomagenesis in immunocompetent host [39].

In conclusion, regarding DLBCL subgrouping by cell of origin, the concordance between GEP and immunohistochemistry data has been poor [29], and the reproducibility of the classification in ABC and GCB subtypes using different immunohistochemical algorithms was also very poor [40, 41]. Therefore, it is important to stress that immunohistochemistry cannot yet be used as a reliable technique for DLBCL classification. Technical standardization on immunohistochemical markers for formalin fixed paraffin-embedded tissue is advisable.

## Genetic landscape of DLBCL NOS

Owing to the application of new technologies (Fig. 2), the ABC and GCB DLBCL subgroups, originally formulated on a cell-of-origin model, have more recently been shown to harbor different pathways of cellular transformation and oncogenesis [42, 43]. In the first study [44] that examined DBLCL with NGS technology, using a combination of whole genome sequencing (WGS), exome sequencing, and RNA sequencing, a recurrent and much targeted somatic mutation affecting the polycomb repressor-2 complex gene EZH2 was identified. EZH2 gene mutation was found in 22 % of DLBCL NOS, all of which were confined to the GCB subgroup. Regarding the ABC subgroup, the major signaling alteration appeared to be the constitutive activation of the nuclear factor kB (NF-kB) pathway through chronic stimulation of the B cell receptor (BCR) pathway [44].

Table 1 summarizes the major discoveries in the genetic landscape of DLBCL NOS using NGS technology [24, 42, 44–60] (reviewed in [7–9, 61–63]). A role for the CBM complex, CARD11, BCL10, and MAL1, downstream of BCR in NF-kB activation, has been demonstrated. Mutations in *CARD11* are observed in approximately 10 % of ABC DLBCLs [42]. A majority of other ABC DLBCLs has been shown to have chronic activation of the BCR pathway through various mechanisms including activating mutations of *CD79A* and *CD79B* and recruitment of Bruton’s tyrosine kinase, which is required for CARD11 signaling [60]. *CREBBP* mutations are observed in 22 % of all DLBCL, with enrichment in the GCB subtype, whereas *E300* mutations are observed in 10 % of all DLBCL [47]. These mutations might be functionally significant in that tumor cells harboring mutant genes could be deficient in acetylating BCL6 and p53, leading to constitutive activation of the BLC6 oncoprotein and to decreased p53 tumor suppressor activity [47]. Importantly, recurrent mutations in several genes affecting histone modification have been identified [48]. A recent study [45] confirmed the above-reported findings [44, 46, 47] identifying *EZH2*, *MYD88*, *CREBBP*, *MLL2*, *MEF2B*, and *CD58*, in addition to several other genes, as targets of recurrent mutation in DLBCL [45]. In summary, the ABC subgroup is particularly linked to activation of the NF-kB pathway. The GCB subgroup of DLBCL is less clearly dependent upon deregulation of a particular pathway.

**Table 1:** Genetic alterations and deregulated signaling pathways in diffuse large B cell lymphoma, not otherwise specified

Cell of origin	Genetic alteration	References	Pathway
GCB like	*BCL2* translocation	Rosenwald et al. [24]	Apoptotic signaling
	*BCL2* mutation	Lohr et al. [45]	Apoptotic signaling
	*EZH2* mutation	Morin et al. [44, 45]	Chromatin remodeling
		Lohr et al. [43]	
	*CREBBP* mutation	Pasqualucci et al.[47]	Chromatin remodeling
		Lohr et al. [45]	
	*TNFRSF14* mutation	Lohr et al. [45]	BCR
	*GNA13* mutation	Morin et al. [48]	
		Lohr et al. [45]	
	*SGK1* mutation	Morin et al. [48]	
	*C-REL* amplification	Rosenwald et al. [24]	NFKB
ABC Not assigned	*BCL6* translocation	Iqbal et al. [49]	
	*INK4/Arf* deletion	Lenz et al. [50]	
	*PRDM1* deletion/mutation	Pasqualucci et al. [51]	
		Pasqualucci et al. [53]	
		Morin et al. [48]	
	*TNFAIP3* deletion/mutation	Compagno et al. [52]	NFKB
		Pasqualucci et al. [53]	
	*SPIB* gain or amplification	Lenz et al. [50]	
	*CARD11* mutation	Lenz et al. [42]	BCR
	*MYD88* mutation	Ngo et al. [46]	BCR
		Lohr et al. [45]	
	*MYC/BCL2* coexpression	Johnson et al. [54]	Apoptotic signaling
		Savage et al. [55]	
	*NFKB* costitutive activity	Davis et al. [59]	NFKB
	*CD79A* mutation	Davis et al. [60]	BCR
	*CD79B* mutation	Davis et al. [60]	BCR
	*CREBBP* mutation	Pasqualucci et al.[47]	Chromatin remodeling
		Lohr et al. [45]	
	*E300* mutation	Pasqualucci et al. [47]	
	*MLL2* mutation	Morin et al.[48]	
		Pasqualucci et al. [53]	
		Lohr et al. [45]	
	*MEF2B* mutation	Morin et al. [48]	
		Lohr et al. [45]	
	*TBL1XR1/TP63* fusion	Scott et al. [56]	
	*NOTCH1* mutation	Lohr et al. [45]	NOTCH
	*NOTCH2* mutation	Lee et al. [57]	NOTCH
	*BRAF mutation*	Lohr et al. [45]	MAPK
	*TP53*	Xu-Monette ZY [58]	Cell cycle regulation

*GCB* germinal center B cells, *ABC* activated B cell

**Fig. 3:**
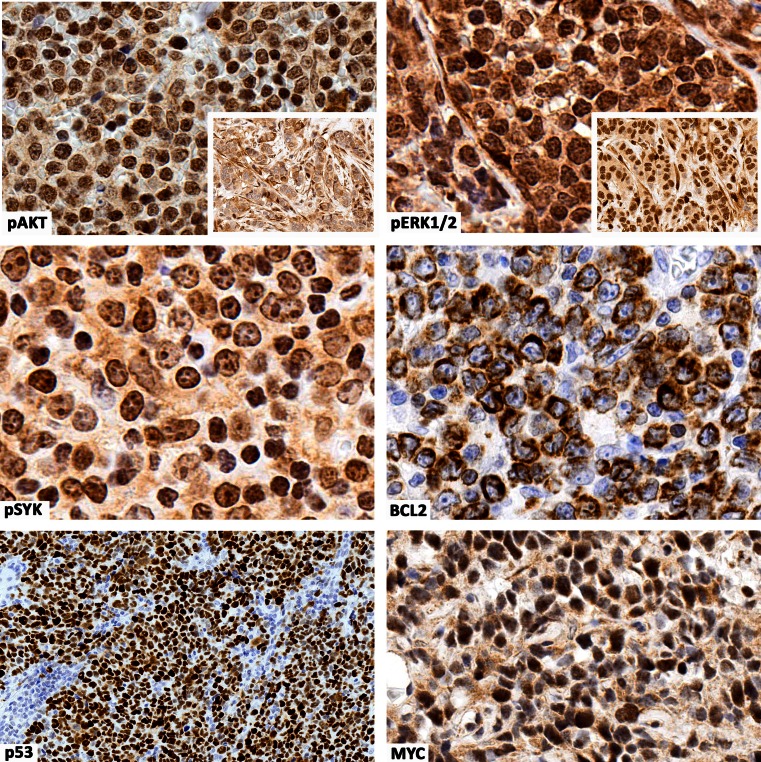
This composite figure shows some examples of immunohistochemical detection of prognostic/potentially predictive biomarker expression in diffuse large B cell lymphoma not otherwise specified (DLBCL NOS). Most tumor cells are immunostained for the various biomarkers tested. Immunostaining for pAKT and pERK1/2 is nuclear and cytoplasmic; similar immunostaining is also observed in breast cancer tumor cells (positive controls, *inset*). Immunostaining for pSYK, p53, and MYC is nuclear, whereas immunostaining for BCL2 is cytoplasmic. Type of specimen: lymph nodes involved by DLBCL NOS. Type of stabilization of specimen: formalin-fixed paraffin-embedded samples. Antibodies: suitable for paraffin-embedded tissues. Images acquired with the Olympus Dot.Slide Virtual microscopy system using an Olympus BX51 microscopy equipped with PLAN APO × 2/0.08 and UPLAN SApo × 40/0.95 objectives

The identification and functional characterization of the molecular bases of deregulated signaling in DLBCL NOS subgroups is providing the preclinical rationale for therapeutic inhibition of the involved pathways [6, 64, 65]. In the light of these discoveries, the next steps should include the recognition of biomarkers related to oncogenic pathways that are deregulated by these gene mutations and the validation of their immunohistochemical detection [66] (Fig. 3 and Table 2). At present, the expression of these biomarkers can be detected by immunohistochemistry, but its performance must increase in some of the protein targets for which antibodies are not ideal yet [67].

**Table 2:** Candidate biomarkers for clinical trials (references [65] and [113] review clinical trials driven by the listed biomarkers) in diffuse large B cell lymphoma, not otherwise specified

Candidate biomarker^a^	Related oncogenic pathway^b^
pSyk, BTK	BCR signaling pathway
pAKT, pan pAKT, pGSK3b, p70S6K, pPRAS40	PI3K pathway
pSTAT3, pSTAT5	JAK/STAT pathway
MDM2, p53	P53 pathway
p65	NFkB pathway
pERK 1/2	MAP kinase pathway
cMYC and BCL2	Apoptotic signaling

^a^Biomarkers for which commercially antibodies are available

^b^Oncogenic pathways in which the listed candidate biomarkers are mainly involved

## Prognostic markers in DLBCL

### p53 expression, MYC deregulation, BCL2 rearrangement, and protein expression in DLBCL (Table 3)

#### TP53

The *TP53* (tumor protein 53) gene encodes the tumor suppressor p53 protein, which plays a crucial part in maintaining genomic stability. p53 exerts transcriptional control on multiple genes involved in cell cycle regulation, DNA repair, and gene transcription [58]. It also interacts with numerous cytosolic proteins associated with the intrinsic mitochondrial apoptosis pathway and autophagy [58]. In p53-deficient mice, malignant lymphoma is the predominant malignancy, occurring in about two thirds of animals [68]. *TP53* dysfunction occurs mostly commonly via mutation in the coding sequence, but aberrations at the gene promoter and gene polymorphisms may also contribute [69]. *TP53* mutation occurs in about 20 % of DLBCL. However, disruption of p53-dependent apoptosis appears to be essential in lymphomagenesis, acting through overexpression of anti-apoptotic proteins including BCL2 and BCL-XL and surviving [58]. Recent data also indicate that a subset of DLBCL harbors a complementary set of alterations of p53 and its downstream cell cycle proteins, collaborating to perturb p53 function leading to lymphomagenesis [70].

**Table 3:** Prognostic markers detectable by routine diagnostic technologies in DLBCL, NOS

Gene/protein	Aberrations	Frequency (%)	Detection	Associated features	Prognosis
TP53 [58, 73]	Mutations, deletion	20	FISH, sequencing, IHC	Large tumor (>7.5–10 cm)	Poor
MYC [55, 76]	Rearrangement, amplification	9–14 (FISH); 30 (IHC)	FISH, IHC	Elderly (>60 years old), high international prognostic index score, advanced stage, extranodal involvement, multiple karyotypic aberrations	Poor
BCL2 [81–83]	Rearrangement, amplification	24–55	FISH, IHC	Advanced stages	Poor
				Marrow involvement	
BCL6 [49, 87]	Rearrangement, hypermutation	55–71	Sequencing, IHC	GCB phenotype	Good
MYC, BCL2 [85]	Rearrangement, amplification	21–29	FISH, IHC	Intermediate between DLBCL and BL double-hit lymphoma	Poor
				Advanced stage	
				High international prognostic index score	

*BL* Burkitt lymphoma, *DLBCL* diffuse large B cell lymphoma, *FISH* fluorescence in situ hybridization, *IHC* immunohistochemistry, *GCB* germinal center B cell, *NPS* not otherwise specified

In general, *TP53* aberrations in DLBCL are associated with poor outcomes, although the exact impact varies somewhat in different studies, owing probably to the adoption of different detection methods, which include immunohistochemical staining for p53, fluorescence in situ hybridization (FISH) for 17p13.1 (which contains *TP53*) deletion, and direct gene mutation analysis. Of these methods, mutational analysis gives the most consistent results. FISH analysis showing loss of heterozygosity (LOH) is less predictive, as LOH needs to collaborate with *TP53* mutation in order to abrogate p53 function. Immunohistochemical analysis is observer dependent, and a pattern of p53 + p21− has been proposed to be more closely associated with *TP53* gene mutation [71].

In the pre-rituximab era, *TP53* mutation had been shown to be a poor prognostic indicator in CHOP-treated cases [72]. The poor prognostic impact still remained in patients treated with ritixumab-CHOP, both for the GCB and ABC subtypes [73]. In addition to prognostic significance, *TP53* perturbations may be a potential molecular marker of DLBCL that are amenable to targeted treatment, particularly with CDK inhibitors [70].

#### MYC

*MYC* was originally identified as a putative oncogene involved in the t(8;14)(q24;q32) translocation in Burkitt lymphoma (BL) [74]. *MYC* rearrangements have subsequently been found in other subtypes of aggressive lymphomas [75]. It is a transcription factor with diverse biologic functions. In oncogenesis, MYC regulates numerous genes involved in cellular proliferation, growth, and DNA replication. By activating CCND2 and other CDKs and suppressing cell cycle inhibitors, MYC promotes transition into S phase. It also regulates many micro-RNAs with oncogenic or tumor suppressor function [74, 75].

*MYC* rearrangements occur in 9–14 % of DLBCL [55, 76]. In contrast to BL, *MYC* rearrangement in DLBCL is often associated with multiple karyotyic aberrations and other genetic lesions [75]. Clinically, patients with *MYC*-rearranged DLBCL were usually >60 years old, presenting with higher IPI scores and more advanced-stage disease, often with extranodal involvement [55, 76]. Accordingly, *MYC* rearrangements portend a poor prognosis in DLBCL treated with standard rituximab-CHOP. In one study, the 5-year overall survival of patients with *MYC* rearrangement was significantly inferior at 33 %, as compared with 72 % in patients without *MYC* rearrangement [55]. In another study, the survival at 2 years of *MYC*-rearranged cases was 35 %, as compared with 61 % in non-rearranged cases [76].

Determination of *MYC* rearrangement with FISH is costly, time-consuming, and not routinely available. Moreover, MYC overexpression may also be due to *MYC* amplification and other cellular mechanisms. The advent of a sensitive and specific monoclonal anti-MYC antibody recognizing the N-terminus of MYC has allowed immunohistochemical detection of nuclear MYC with high accuracies in paraffin-embedded sections [77]. Cases with *MYC* rearrangement show strong nuclear MYC staining (>70 % of cells) [77]. However, MYC overexpressing cases may not always harbor *MYC* rearrangement, suggesting alternative mechanisms of upregulation of MYC.

#### BCL2

*BCL2* is an oncogene originally identified from the t(14;18)(q32;q21) translocation found in follicular lymphoma [78]. Overexpression of BCL2 leads to extended B cell survival and follicular lymphoproliferation in transgenic mice, recapitulating the human disease [78]. It is an anti-apoptosis protein important in normal B cell development and differentiation. BCL2 overexpression provides a survival advantage to neoplastic B cells and may play a part in resistance to chemotherapy [79].

BCL2 overexpression can be related to *BCL2* rearrangement or other cellular mechanisms. Early immunohistochemical studies coupled with FISH analysis had shown BCL2 overexpression in about 24–55 % of cases of DLBCL [80–82]. BCL2 overexpression tended to be associated with advanced stage and inferior survivals. However, the results were not always consistent. These conflicting findings were partly resolved, when later studies showed that the prognostic significance of BCL2 might vary depending on the cellular context. With immunohistochemical analysis, it was observed that BCL2 expression in GCB DLBCL was associated with t(14;18) and did not correlate with prognosis. However, BCL2 expression in ABC DLBCL was not associated with t(14;18), but often with *BCL2* gene amplification and activation of the NFKB pathway, and portended inferior survivals [83]. These findings were also confirmed by FISH analysis of *BCL2* rearrangement, which was found to correlate strongly with GCB phenotype, but did not significantly impact on survivals [84].

#### Complex genetic alterations involving MYC, BCL2, and BCL6

The prognostic impact of *MYC* and *BCL2* rearrangement and overexpression taken individually appears significant in DLBCL, but is not always unequivocal. Emerging data indicate that rearrangement and overexpression of *MYC* and *BCL2* may collaborate to negative-effect survivals.

In earlier studies, where FISH was used to analyze *MYC* and *BCL2* rearrangements, DLBCL with concomitant *MYC*/*BCL2* rearrangements had dismal survivals, with about 60 % of patients dying within 6 months, and a 5-year survival of less than 10 % [54]. Notably, about two thirds of these “double-hit” lymphomas were classified as B cell lymphoma unclassifiable, with features intermediate between DLBCL and BL. These observations were further extended with routine immunohistochemical analysis of MYC and BCL2 overexpression. In one study, MYC+/BCL2+ cases were found to constitute 21 % of 167 cases of DLBCL [85]. MYC protein overexpression (in >40 % of neoplastic cells) predicted inferior survivals with rituximab-CHOP only when BCL2 protein was concomitantly overexpressed (>50 % of neoplastic cells). Although MYC protein overexpression correlated strongly with *MYC* rearrangement, actual concomitant *MYC*/*BCL2* rearrangement occurred in 5 % of cases, which was associated also with a dismal prognosis. In another study, 29 % of 193 cases of DLBCL were MYC+/BCL2+ on immunohistochemical evaluation. These patients had inferior response rates, overall survival, and disease-free survivals, which were independent of IPI scores and GBC/ABC origins. Consistently, on FISH analysis, the negative prognostic impact of *MYC* rearrangement was only evident when *BCL2* was also rearranged [86]. Recent data on a large cohort of DLBCL treated uniformly with rituximab-CHOP also confirmed the negative prognostic relevance of the MYC+/BCL2+ phenotype, with concomitant *TP53* mutation conferring an even worse prognosis in such cases [63]. These studies have shown some salient features. The poor outcome of MYC + DLBCL is largely the result of concurrent BCL2 overexpression, and it is the concomitant MYC+/BCL2+ phenotype that predicts outcome. Immunohistochemistry is significantly more sensitive than FISH in defining MYC and BCL2 overexpression. The MYC+/BCL2+ phenotype occurs both in GCB and ABC DLBCL, suggesting that molecular pathways accounting for MYC and BCL2 overexpression might be heterogeneous. Although some of the cases are morphologically B cell lymphoma with features intermediate between DLBCL and BL, histopathologic evaluation is inadequate in predicting the MYC+/BCL2+ phenotype. Finally, patients have a high median age of nearly 70 years, suggesting that MYC+/BCL2+ cases may be a disease predominantly of the elderly population.

Recent data have indicated a possible third player *BCL6* in impacting on prognosis in DLBCL. *BCL6* was originally identified from chromosomal breakpoints at 3q27 [87]. BCL6 is expressed predominantly in GCB cells and is essential for GC formation. In DLBCL, BCL6 is deregulated by gene translocation or somatic hypermutation. Early studies had shown that high *BCL6* gene expression was associated with a better outcome [88]. *BCL6* expression occurred predominantly in GCB DLBCL [49], which might explain the more favorable outcome. In a recent study of elderly patients treated with rituximab-CHOP, low BCL6 expression (<25 % of neoplastic cells) as defined by immunohistochemistry was shown in concert with high BCL2 expression to impart an unfavorable outcome to cases with *MYC* rearrangement or overexpression, independent of the IPI scores [89]. Hence, a “triple-hit” phenotype of MYChigh, BCL2high, and BCL6low has been proposed to confer an even worse prognosis in DLBCL than MYC+/BCL2+ cases.

While these results have shown that immunohistochemical staining for MYC, BCL2, and BCL6 is robust, can be performed routinely, and preferred over FISH analysis, the reproducibility of the defined cutoff has to be defined. However, the double-hit or triple-hit status of DLBCL obviously has to be determined in future trials of DLBCL, particularly when novel agents are tested. Outside clinical trials, it is still unclear how information on MYC, BCL2, and BCL6 may be used in the treatment of individual patients in order to improve outcome. Rituximab-CHOP does not appear to be satisfactory treatment for these cases. As many of these patients are elderly, aggressive chemotherapy or hematopoietic stem cell transplantation may not be feasible. It remains to be defined if therapy targeted against MYC or BCL2 may be valid therapeutic options.

### Microenvironmental, viral, and host’s factors influencing DLBCL prognosis

Retrospective studies have confirmed the worse prognosis for patients with DLBCL ABC subgroup. However, when multivariate analyses are used, it is clear that some of the differences may well be related to older age at diagnosis and other adverse presentation features [34]. In fact, in addition to cytomorphology, immunophenotype, and molecular characteristics derived from newer tools, DLBCL prognosis may be influenced by diverse factors such as the “stromal signature” of the background, viral infections, and host’s factors (age and site) (reviewed in [26]). Moreover, CD5-positive DLBCL has been suggested in some studies, mainly from Japan, to have distinct clinical features [90]. These patients are often older and present with bulky retroperitoneal disease. These cases arise de novo and have no relation to other lymphomas which express CD5, i.e., chronic lymphocytic leukemia or mantle cell lymphoma. Ki-67 index has been reported to be a prognostic marker [91]. A high proliferation rate, as assessed by immunohistochemistry with anti-Ki67 antibody, has been associated with adverse outcome [2]. Coherently, the proliferation signature by one gene expression proved to be a strong predictor of poor survival [24]. In the pre-rituximab era, the IPI, which is based on clinical parameters, such as age, stage, serum lactate deydrogenases level, and performance status, an extent of extranodal involvement proved to be highly valuable for the prediction of prognosis in patients with DLBCL [92]. However, the IPI seems to have lost some of its high predictive value in the rituximab era [93]. Very recently, the so-called enhanced IPI, an updated version of the IPI for patients with DLBCL treated in the rituximab era, has been proposed [94]. Table 4 highlights highly aggressive DLBCL subgrouped according to adverse prognostic factors: lack of CD20 expression, special phenotypes linked to cell of origin and CD5 expression, EBV infection, and complex karyotypes [95, 96].

**Table 4:** Special phenotipes (CD20−, CD5+), complex genotypes, and EBV infection are adverse factors in DLBCL, NOS, and in specific DLBCL subtypes

CD20 negative DLBCL
Plasmablastic lymphoma
Primary effusion lymphoma
ALK-positive large B cell lymphoma
Molecular and immunohistochemical subgroups
Activated B cell like (non-GCB)
CD5+ DLBCL
EBV-related DLBCL
EBV+, DLBCL of the elderly
DLBCL associated with chronic inflammation
Lymphomatoid granulomatosis
Plasmablastic lymphoma
HHV8-/KSHV-positive lymphomas
Unclassifiable/intermediate with genetic/caryotypic complexity
B cell lymphoma, unclassifiable with features intermediate between DLBCL and Burkitt lymphoma
B cell lymphoma, unclassifiable with features intermediate between DLBCL and Hodgkin lymphoma

*DLBCL* diffuse large B cell lymphoma, *GCB* germinal center B cell, *EBV* Epstein Barr virus, *HHV8/KSHV* human herpesvirus 8/Kaposi sarcoma-associated herpesvirus, *NOS* not otherwise modified

#### The microenvironment in DLBCL

The microenvironment and the inflammatory response may provide clues to the behavior of DLBCL according to two patterns of stromal signature predictive of good survival, “stromal 1” (including genes encoding for extracellular matrix proteins), and poor outcome, “stromal 2” (including angiogenetic switch-related genes) [97]. A recent study on the tumor microenvironment and viral components has shown that DLBCL occurring in HIV-infected patients is highly angiogenic with markedly higher blood-vessel density than sporadic DLBCL cases. Importantly, the investigation has also highlighted the role of Epstein Barr virus (EBV) in angiogenesis [98].

#### DLBCL associated with immune dysfunction and infectious agents

DLBCL associated with infectious agents include the spectrum of HIV- and gamma herpesviruses-associated lymphomas and are highly aggressive tumors (Table 5) [39, 99, 100]. EBV-associated lymphomas that are related to chronic inflammation or senescence of the immune system represent distinct disease entities occurring in non AIDS-related settings.

**Table 5:** Virus-associated lymphomas assessed by the IARC Monographs Working Group [39]. The table highlights DLBCL, NOS

Group 1 agent	Lymphomas on which *sufficient evidence* in humans is based	Other lymphomas with limited evidence in humans	Established mechanistic events
Epstein–Barr virus (EBV)	Burkitt lymphoma; immune-suppression-related non-Hodgkin lymphoma, *including DLBCL*, *NOS*, and plasmablastic lymphomas; extranodal NK/T-cell lymphoma (nasal type), Hodgkin lymphoma	–	Cell proliferation, inhibition of apoptosis, genomic instability, cell migration
Hepatitis B virus (HBV)	–	Non-Hodgkin lymphoma,^a^ *including DLBC*, *NOS*	Inflammation, liver cirrhosis, chronic hepatitis
Hepatitis C virus (HCV)	Non-Hodgkin lymphoma,^a^ *including DLBCL*, *NOS*	–	Inflammation, liver cirrhosis, liver fibrosis
Kaposi sarcoma herpes virus (KSHV)	Primary effusion lymphoma^a^	–	Cell proliferation, inhibition of apoptosis, genomic instability, cell migration
Human immunodeficiency virus, type 1 (HIV-1)	Non-Hodgkin’s lymphoma, *including DLBCL*, *NOS*; Hodgkin lymphoma^a^	–	Immunosuppression (indirect action)

*DLBCL*, diffuse large B cell lymphoma; *NOS*, not otherwise specified

^a^Newly identified link between virus and cancer. Modified and adapted from ref. [99]

#### DLBCL associated with chronic inflammation

DLBCL associated with chronic inflammation most commonly involves body cavities. The prototype for this category is pyothorax-associated lymphoma (PAL)[101, 102]. Other cases of DLBCL occurring in the setting of chronic inflammation (such as chronic skin ulcers or osteomyelitis) are also frequently positive for EBV (reviewed in [26]).

#### EBV-positive DLBCL of the elderly

EBV-positive DLBCL of the elderly, also known as age-related or senile EBV-associated lymphoproliferative disorders [103], is diagnosed in patients older than 50 years with no known cause of immunodeficiency or prior lymphoma [103]. Seventy percent of these patients present with extranodal involvement and >50 % have advanced disease with poor prognosis and a short survival rate.

#### DLBCL based on an anatomic site

Some peculiar DLBCL subtypes are specifically related to their sites of presentation. Examples include primary cutaneous DLBCL, “leg type” [104], primary mediastinal (thymic) large B cell lymphoma [105–107], DLBCL of the central nervous system [108, 109], and primary large B cell lymphomas of bones [110, 111]. These DLBCL subtypes express BCL2 and MUM1/IRF4 but not CD10, are frequently related to an ABC phenotype, and are distinct entities with an aggressive behavior [112].

## Biomarkers enrichment strategies to guide therapy

At the molecular level, DLBCL is a heterogeneous disease. Both GEP studies and DNA sequencing studies have demonstrated that DLBCL can be further subdivided into smaller, more homogeneous groups [6–9]. These findings not only explain why patients respond differently to a specific therapy but also provide an opportunity for designing tailored treatments based on tumor characteristics. Such characteristic biomarkers will need to be reproducible across different laboratories, and preferentially, should guide therapeutic options.

**Fig. 4:**
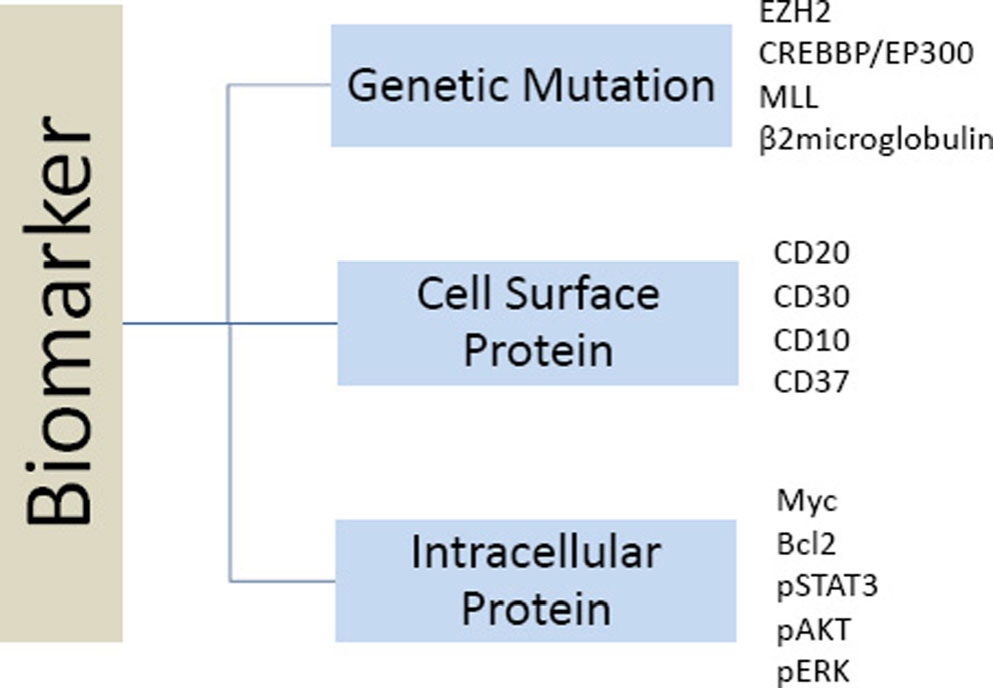
Genetic and protein biomarkers can be used to stratify patients for new treatment strategies. The figure lists a set of biomarkers including genetic mutations, cell surface proteins, and intracellular proteins.

In recent years, GEP methods were used to stratify patients based on the cell of origin, into GCB or ABC DLBCL. While this approach helped advancing the field and patient selection for clinical trials, it continues to lack mechanistic precision. Both genetic and protein biomarkers can be used to more precisely stratify patients for evaluating novel treatment regimens based on molecular mechanisms that support tumor growth and survival. However, at the present time, the ideal biomarker or biomarker set remains elusive. For this reason, it is advised to include several biomarkers as part of correlative discovery biomarker analysis. As shown in Fig. 4, a biopsy can be analyzed for a set of biomarkers: genetic mutations, cell surface proteins, and intracellular proteins. Other biomarkers can also be included, such as gene expression status. Regardless, it would be more efficient to screen patient tumors for several biomarkers at the same time using an umbrella protocol. For example, a lymphoma specimen can be examined for the presence of genetic alterations using targeted sequencing strategies. At the same time, the biopsy can be examined for several immunohistochemistry-based biomarkers, such as the cell of origin, MYC and BCL2 expression, and the expression of phosphoproteins that are associated with activated oncogenic signaling pathways, such as pSTAT3, pERK, and pAKT. Based on this analysis, patients can be offered targeted agents that match and are more suitable for their tumors. Thus, in an “umbrella” clinical trial, several targeted agents can be simultaneously tested in biomarker-defined populations. However, when a tumor expresses several biomarkers at the same time, a prioritization algorithm should be implemented. For example, if a lymphoma specimen is shown to have EZH2 mutation and pSTAT3 expression, experimental treatment with an EZH2 inhibitor or a JAK2 inhibitor can be offered, but which treatment should be considered first? Eventually, the coexpression of more than one targeted biomarker may offer an opportunity to guide new treatment strategies [64, 113]. For example, a lymphoma specimen that coexpresses high levels of MYC and BCL2 proteins may respond better to a combination regimen that targets both proteins. The selection of such combination regimens should be based on synergistic effects in preclinical studies and should first be evaluated in phase 1 trials to establish its safety in the clinical setting.

Because many genetic mutations in DLBCL occur at a very low frequency, it would be more efficient to group several mutations into one mechanistic category, such as activation of well-defined oncogenic signaling pathways [65]. Such a strategy will require linking signaling pathways with a unifying set of biomarkers that can be detected by immunohistochemistry. For example, mutations in PI3K, AKT, TSC, mTOR, deletion of PTEN, and phosphorylate PRAS40 protein may be grouped into one bucket of “activated PI3K pathway.” Mechanism-based treatment strategies can then be evaluated to inhibit different components in this pathway, rather than targeting each genetic mutation separately. This strategy can be more efficient for enriching patients for specific trials.

## Concluding remarks

The era of treating all patients with DLBCL with the same regimen is fading away. New strategies of “divide and concur” are gaining momentum as it divided patients into several groups based on their tumor characteristics. Identifying these patients through clinical biomarkers is now feasible and may allow in the future the administration of more precise therapy for different patients. These strategies are currently being evaluated in clinical trials. For example, bortezomib and ibrutinib are in trials for patients affected by ABC DLBCL, while EZH2 and BCL6 inhibitors may be used for other patients affected for GCB DLBCL. Patient participation in these clinical trials is critical for expediting our progress in improving the cure rate of patients with DLBCL.
